# Evaluation of microRNA expression in plasma and skeletal muscle of thoroughbred racehorses in training

**DOI:** 10.1186/s12917-017-1277-z

**Published:** 2017-11-22

**Authors:** B. A. McGivney, M. E. Griffin, K. F. Gough, C. L. McGivney, J. A. Browne, E. W. Hill, L. M. Katz

**Affiliations:** 1 UCD School of Agriculture and Food Science, Section of Animal & Crop Sciences, University College Dublin, Belfield, Dublin 4, Ireland; 2 UCD School of Veterinary Medicine, Section of Veterinary Biomedical Sciences, University College Dublin, Belfield, Dublin 4, Ireland

**Keywords:** Horse, miRNA, Exercise, Plasma, Haemolysis, Skeletal muscle

## Abstract

**Background:**

Circulating miRNAs (ci-miRNAs) are endogenous, non-coding RNAs emerging as potential diagnostic biomarkers. Equine miRNAs have been previously identified including subsets of tissue-specific miRNAs. In order to investigate ci-miRNAs as diagnostic tools, normal patterns of expression for different scenarios including responses to exercise need to be identified. Human studies have demonstrated that many ci-miRNAs are up-regulated following exercise with changes in expression patterns in skeletal muscle. However, technical challenges such as haemolysis impact on accurate plasma ci-miRNA quantification, with haemolysis often occurring naturally in horses following moderate-to-intense exercise. The objectives of this study were to identify plasma ci-miRNA profiles and skeletal muscle miRNAs before and after exercise in Thoroughbreds (Tb), and to evaluate for the presence and effect of haemolysis on plasma ci-miRNA determination. Resting and post-exercise plasma ci-miRNA profiles and haemolysis were evaluated in twenty 3 year-old Tbs in sprint training. Resting and post-exercise skeletal muscle miRNA abundance was evaluated in a second cohort of eleven 2 year-old Tbs just entering sprint training. Haemolysis was further quantified in resting blood samples from twelve Tbs in sprint training. A human plasma panel containing 179 miRNAs was used for profiling, with haemolysis assessed spectrophotometrically. Data was analysed using a paired Student’s *t*-test and Pearson’s rank correlation.

**Results:**

Plasma ci-miRNA data for 13/20 horses and all skeletal muscle miRNA data passed quality control. From plasma, 52/179 miRNAs were detected at both time-points. Haemolysis levels were greater than the threshold for accurate quantification of ci-miRNAs in 18/25 resting and all post-exercise plasma samples. Positive correlations (*P* < 0.05) between haemolysis and miRNA abundance were detected for all but 4 miRNAs, so exercise-induced changes in plasma ci-miRNA expression could not be quantified. In skeletal muscle samples, 97/179 miRNAs were detected with 5 miRNAs (miR-21-5p, let-7d-3p, let-7d-5p, miR-30b-5p, miR-30e-5p) differentially expressed (DE, *P* < 0.05) between time-points.

**Conclusions:**

The degree of haemolysis needs to be determined prior to quantifying plasma ci-miRNA expression from horses in high-intensity exercise training. Identification of DE miRNAs in skeletal muscle indicates modification of miRNA expression may contribute to adaptive training responses in Tbs. Using a human plasma panel likely limited detection of equine-specific miRNAs.

## Background

MicroRNAs (miRNAs) are endogenous, non-coding RNAs with complex roles causing translational repression/degradation of bound mRNA [1], regulating many cellular processes [2]. They have intracellular effects, are extremely stable when secreted into the bloodstream [3] and have been identified in multiple body fluids [4]. MiRNAs are often tissue-specific and can be secreted from their tissue of origin into the bloodstream bound to lipoproteins [5] or packaged into extracellular microparticles [6]. When in blood they are termed circulating miRNAs (ci-miRNAs) [7] and are emerging as potential diagnostic biomarkers due to their stability [8] and ease of access for sample collection.

Human studies have identified a multitude of ci-miRNAs up-regulated following intense exercise training [9–12]. Furthermore, exercise training has been found to alter the expression pattern of miRNAs in skeletal muscle [11, 13, 14]. It has been hypothesised that skeletal muscle may contribute to changes in differentially expressed (DE) ci-miRNAs in response to exercise training by releasing exosomes into plasma [15, 16], supporting the possibility that DE plasma ci-miRNAs could be used to assess adaptive training responses of skeletal muscle.

MiRNA analysis in horses is an emerging area of research. To-date, >700 equine miRNAs have been identified with subsets of tissue-specific DE miRNAs isolated [17] including skeletal muscle and blood [18, 19]. Recently, 197 miRNAs were identified in equine skeletal muscle, with 76 found to be muscle-specific [20]. However, to-date there has only been one study evaluating ci-MRNA in exercising horses [21]. In this report, 167 DE miRNAs were identified from pre- and post-endurance exercise blood samples; however, whole-blood rather than plasma/serum was evaluated making it difficult to determine if ci-miRNAs were expressed from red blood cells (RBC) or other tissue sites such as skeletal muscle.

There are technical challenges involved in plasma/serum ci-miRNA measurement such as inaccurately quantifying plasma RNA due to low yields, haemolysis impacting on ci-miRNA quantification and a lack of validated stable reference miRNAs [22–25]. Since haemolysis may occur naturally in horses following moderate-to-intense exercise [26, 27], this needs to be considered when attempting to evaluate equine plasma/serum ci-miRNA expression.

The aim was to identify DE plasma ci-miRNAs and skeletal muscle miRNAs before and after exercise in Thoroughbreds (Tb), and to evaluate for the presence and effect of haemolysis on plasma ci-miRNA determination.

## Methods

A Department of Health License (B100/3525), ethical approval from the University College Dublin Animal Research Ethics Committee and owner consent were obtained for all horses and procedures in this study.

### Sample population

All horses in the study were from the same stable and were managed and trained in a similar way [28]. Horses trained 6 days per week with gradual introduction of 800–1000 m sprint training (work day [WD]) alternating with submaximal training, following which horses entered competitive racing.

### Experimental protocol

#### Cohort A: Plasma ci-miRNA

Plasma lactate concentrations ([LA]) and ci-miRNA were evaluated in *n* = 20 sex- and fitness-matched 3 year-old Tbs (*n* = 10 males, *n* = 10 females) in active training and racing before (T_0_) and 5 mins after (T_5min_) a WD training session. The degree of haemolysis was evaluated in all samples.

#### Cohort B: Skeletal muscle miRNA

Resting (T_0_) and T_5min_ plasma [LA] along with T_0_ and 4 h (T_4hr_) post-exercise skeletal muscle miRNAs were evaluated in *n* = 11 2 year-old Tbs (*n* = 7 males, *n* = 4 females) before and after their first WD training session.

#### Cohort C: Degree of plasma haemolysis

The degree of haemolysis was further evaluated in T_0_ plasma samples from an additional *n* = 12 Tbs in active training and racing (*n* = 5 males, *n* = 7 females, 2–4 years old).

### Data collection

Prior to an exercise test horses were fitted with a heart rate (HR) telemetry system^1^ and GPS unit^2^ which recorded speed, HR and distance.

Resting jugular venous blood samples were collected before feeding and exercise. Samples for plasma [LA] and ci-miRNA measurement were placed into fluoride oxalate and ethylenediamine tetraacetic acid (EDTA) tubes, respectively. Once collected all blood tubes were immediately centrifuged with plasma separated. [LA] was measured on-site with an autoanalyser.^3^ Plasma for haemoglobin concentrations [Hb] and ci-miRNA analysis was stored at 4 °C until transported to be stored at -80 °C (within 6 h of collection) and analysed as a batch at a later date.

### Blood samples for miRNA

MiRNAs were extracted from plasma samples^4^ with 4 μL amounts then reversely transcribed in 20 μL reactions.^5^ Exogenous RNA spike-ins (UniSp2, UniSp4, UniSp5, cel-miR-39-3p) provided by the manufacturer of the human serum/plasma focus miRNA PCR panel kit were added during RNA extraction and analysed according to the manufacturer’s instructions^5^.

### Haemolysis analysis

The degree of haemolysis was assessed by quantifying plasma [Hb] using a UV/VIS spectrophotometer,^6^ measuring oxyhaemoglobin absorbance at k = 414 nm [22]. A value of ≤0.2 absorption units (AU) was used as the threshold for acceptable haemolysis and accurate quantification of ci-miRNA detection [22].

### Skeletal muscle biopsy samples for miRNA

Skeletal muscle biopsies were obtained from the *gluteus medius* using a 6 mm-diameter, modified Bergstrom biopsy needle^7^ according to previously described methods [29]. Samples were preserved in RNAlater^8^ until further processing.

Total RNA was extracted from 50 to 100 mg of tissue using a protocol combining TRIzol reagent,^9^ DNase treatment^10^ and RNeasy.^11^ RNA was quantified using a Qubit fluorimeter with the Qubit RNA HS assay. RNA quality was assessed using the 18S/28S ratio and RNA integrity number on an Agilent Bioanalyzer with the RNA 6000 Nano Lab-Chip kit.^12^ RNA (2 μL@5 ng/ul) was reversely transcribed in 10 μL reactions^5^. UniSp6 (exogenous RNA spike-in) was added during cDNA conversion.

### Real-time quantitative polymerase chain reaction measurements

cDNA was assayed in 10 μL PCR reactions^5^. All miRNAs were assayed once by RT-qPCR on the Exiqon Human Serum/Plasma Focus miRNA PCR panel I and II. Each panel consisted of a 96 well plate with 179 miRNAs of interest and 15 controls (inter-plate calibrators, negative and spike-in controls). Amplification was performed in an Applied Biosystems StepOnePlus™ Real-Time PCR System. Amplification curves were analysed using Applied Biosystems software to obtain raw PCR cycle threshold (*C*_t_) values.

### miRNA quantification

Quality control measures (inter-plate calibration, ommission of outliers, miRNAs with *C*_t_ values >34 from analysis) were carried out using GeneEx software. MiRNAs with >80% missing data were removed from the analysis, with missing values replaced with the mean value for that miRNA. Results from muscle biopsies were normalised to the global mean.

### Statistical analysis

A paired Student’s *t*-test was used to compare T_0_ and T_5min_ plasma haemolysis and ci-miRNAs and T_0_ and T_4h_ muscle miRNA abundance_._ A Shapiro-Wilk test was performed to confirm that the *C*_t_ data was normally distributed and the Benjamini and Hochberg method used to correct for multiple testing. Pearson’s rank correlation was used to evaluate correlation between T_0_ and T_5min_ ci-miRNA expression level and absorbance at 414 nm, and a paired Student’s *t*-test used to assess the significance of that correlation. Significance was set at *P* ≤ 0.05.

### Prediction and annotation of miRNA targets

Prediction of potential target genes and pathway analysis was completed using the DIANA-mirPath software [30] in the Diana Tools suite. The DIANA-microT-CDS algorithm was used to predict potential target genes for DE miRNAs; these were then utilised by miRPath for KEGG pathway analysis, enriched through analysis of all predicted targets. A 0.8 default setting was used as the MicroT prediction score threshold with species set to human since there was no equine option. Since there is a high conservation of miRNAs accross species suggesting the sharing of biological functions, it was believed that inferences could be made on the function of equine miRNAs based on their human homologues. Significance for over-represented KEGG pathways was set as *P* ≤ 0.05 following Benjamini and Hochberg correction for multiple testing.

## Results

### Quality control for miRNA quantification

The miRNA data for 7/20 horses from cohort A failed quality control and were omitted from analysis. All samples from cohort B passed quality control.

### Physiological exercise parameters

For all horses undertaking an exercise test in the study, the average V_peak_ was 16.9 ± 0.98 m/s (range 15.2–18.5), the average HR_peak_ 221.4 ± 7.1 bpm (range 209–232) and the average total exercise distance 1130.19 ± 145.1 m/s (range 876–1471). The average pre- and 5 mins post-exercise plasma [LA] were 0.46 ± 0.09 mmol/l (range 0.33–0.63) and 26.3 ± 3.68 mmol/l (range 19.2–32.1), respectively. There were no significant differences between males and females regarding any of the exercise and physiological measurements.

#### Cohort A: Plasma ci-miRNA

**Fig. 1:**
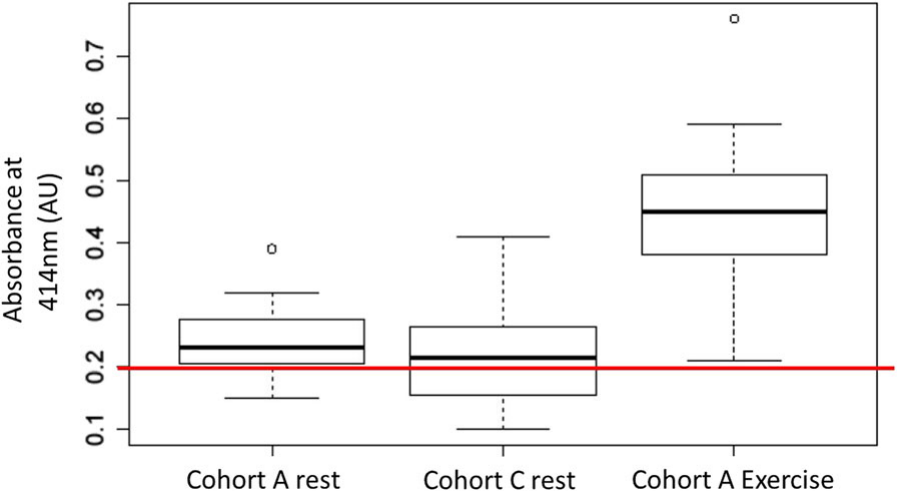
Box and whiskers plot depicting the degree of haemolysis based on absorbance at 414 nm for *n* = 13 equine plasma samples before and after exercise (cohort A) and from *n* = 12 equine plasma sampes from horses at rest (cohort C). A value of ≤0.2 absorption units was used as the threshold for acceptable haemolysis and accurate quantification of ci-miRNA detection. The red line indicates the threshold above which accurate quantification of ci-miRNA is not possible

**Fig. 2:**
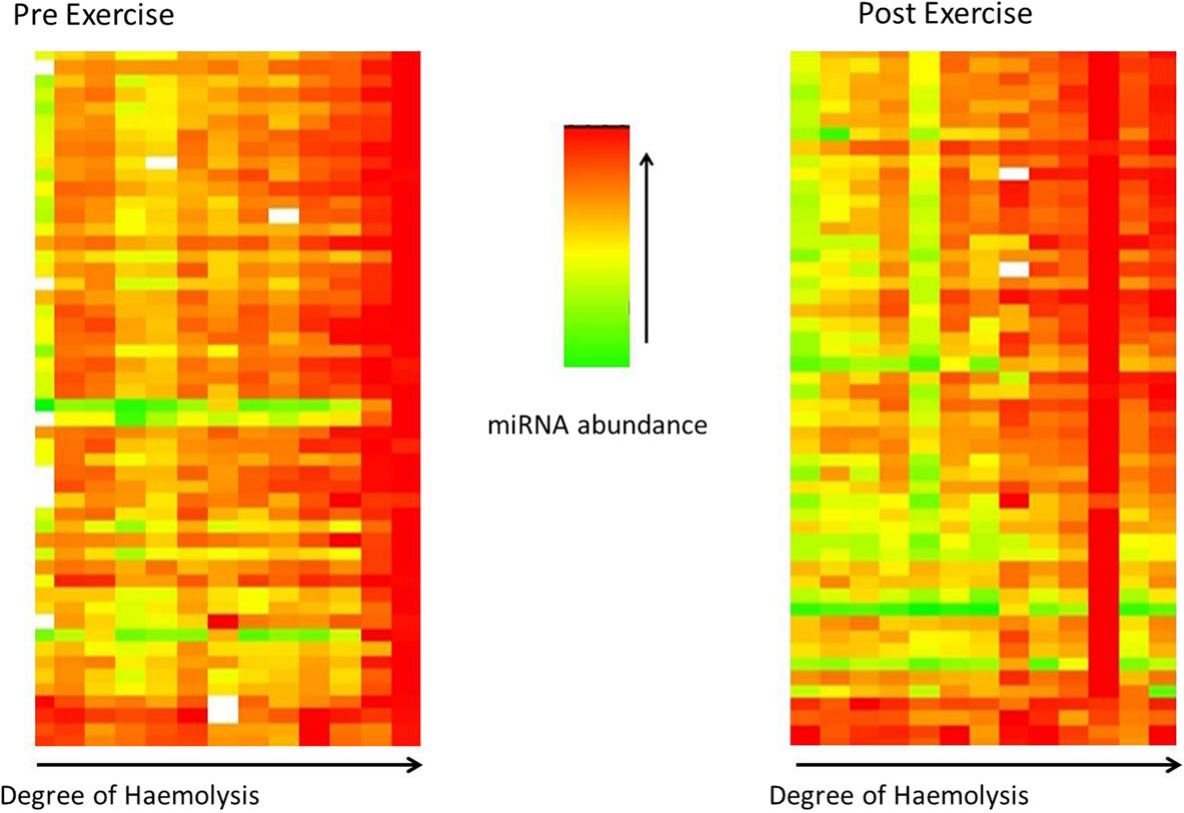
Heatmap showing the levels of 52 miRNAs relative to the degree of haemolysis from *n* = 13 equine plasma samples before and after exercise. MiRNA abundance is presented as *C*_t_ values and degree of haemolysis is the absorbance at 414 nm for each plasma sample

In the equine plasma samples, 29% (52/179) of the miRNAs on the human plasma panel were detected at both timepoints, all of which had increased expression (3.23 ± 1.5 fold mean increase, 1.1–10.3 range increase) at T_5min_ when compared to T_0_. However, absorbance >0.2 AU at 414 nm were detected in 11/13 T_0_ and all T_5min_ plasma samples for horses in cohort A, with absorbance peaks significantly higher in the T_5min_ samples (*P* = 1.2^−05^; Fig. 1). The T_0_ absorbance ranged from 0.15–0.39 AU with an average of 0.25 ± 0.06 AU while the T_5min_ values ranged from 0.26–0.76 AU with an average of 0.46 ± 0.13 AU. The expression levels of 35 miRNAs were significantly correlated (*r* > 0.55, *P* < 0.05) with absorbance and thus haemolysis levels in both T_0_ and T_5min_ samples while 13 miRNA expression levels were only significantly correlated with haemolysis in T_0_ samples; 4 miRNAs were not correlated with haemolysis at either time point. Due to the high levels of haemolysis in the plasma samples and the strong positive correlation between haemolysis and miRNA abundance (Fig. 2), it was not possible to accurately quantify exercise-induced changes in plasma ci-miRNA expression in the present study. There were no significant differences between males and females regarding any of the haemolysis and ci-miRNA measurements.

#### Cohort B: Skeletal muscle miRNA

**Fig. 3:**
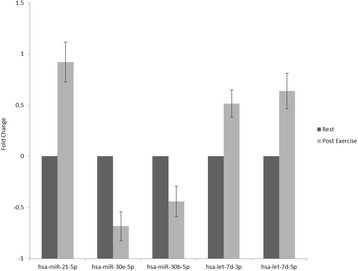
Real time qRT-PCR results for miRNAs differentially expressed in skeletal muscle before and following exercise from *n* = 11 Thoroughbred horses. The standard 2-ΔΔCT method was used to determine mean fold changes in gene expression. All *C*t values were normalised using the global normalisation function in the GeneEx software package. A Student’s *t*-test was used to identify significant differences in mRNA abundance between time-points

In the equine skeletal muscle biopsy samples, 55% (98/179) of the miRNAs on the human plasma panel were detected at both T_0_ and T_4h_. Following the Benjamini and Hochberg correction for multiple testing, 5 of these miRNAs were significantly DE at T_4h_ (*P* < 0.05; Fig. 3). The greatest expression difference was detected in miRNA miR-21-5p which increased 0.92 ± 0.2 fold. The expression of the miR-30b-5p and miR-30e-5p family members increased 0.52 ± 0.1 and 0.64 ± 0.2 fold, respectively. Expression of let-7d-3p and let-7d-5p decreased 0.68 ± 0.1 and 0.44 ± 0.1 fold, respectively. Using the DIANA-microT-CDS algorithm a total of 1687 gene targets for these 5 miRNAs were identified. The largest number of gene targets were identified for the miR-30 family members. In total 1077 of the predicted targets were unique to these 2 miRNAs while only 3 gene targets were predicted for let-7d-3p. The most over-represented KEGG pathways for each miRNA based on a predicted target gene list are summarised in Table 1 [31, 32]. Pathways previously implicated in the equine exercise response were significantly enriched among predicted targets of the DE miRNAs (Table 2). These included the Jak-STAT signaling, MAPK signaling, insulin signaling and mTOR signaling pathways and long-term potentiation.

**Table 1:** The most over-represented KEGG pathways for each miRNA based on a predicted target gene list

miRNA	Fold change	adjusted *P* value	*n* genes	Top KEGG pathway	*P* value
hsa-let-7d-3p	−0.68	0.002	3	NA	NA
hsa-miR-21-5p	0.92	0.002	243	Cytokine-cytokine receptor interaction	2.48E-06
hsa-let-7d-5p	−0.44	0.022	370	ECM-receptor interaction	9.22E-06
hsa-miR-30b-5p	0.52	0.023	1113	Ubiquitin mediated proteolysis	4.76E-10
hsa-miR-30e-5p	0.64	0.042	1153	Ubiquitin mediated proteolysis	2.92E-08

**Table 2:** Pathways previously implicated in the equine exercise response that were significantly enriched among predicted targets of the differntially expressed miRNAs

KEGG pathway	*P* value	*n* genes	*n* miRNAs
B cell receptor signalling pathway	1.4637E-06	16	4
Ubiquitin mediated proteolysis	1.4637E-06	25	4
Neurotrophin signalling pathway	3.75787E-06	22	4
T cell receptor signalling pathway	3.75787E-06	20	4
TGF-beta signalling pathway	9.00943E-06	15	4
PI3K-Akt signalling pathway	4.61509E-05	43	4
Long-term potentiation	0.000171436	13	4
Regulation of actin cytoskeleton	0.000171436	28	4
MAPK signalling pathway	0.000293552	34	4
Jak-STAT signalling pathway	0.000485158	22	4
Axon guidance	0.000671812	22	3
Arrhythmogenic right ventricular cardiomyopathy	0.000834821	13	3
Hepatitis B	0.000838527	21	4
Transcriptional misregulation in cancer	0.002038503	26	4
Hypertrophic cardiomyopathy	0.002113538	13	3
Insulin signalling pathway	0.002113538	19	4
Mucin type O-Glycan biosynthesis	0.002113538	6	3
Prostate cancer	0.0021797	13	4
mTOR signalling pathway	0.002349162	11	4
Acute myeloid leukaemia	0.003086604	9	4
Adherens junction	0.004193219	14	4

#### Cohort C: Degree of plasma haemolysis

The average absorbance at 414 nm for this cohort was 0.21 ± 0.09 AU, ranging from 0.10–0.41 AU (Fig. 1); 7/12 samples had absorbtion peaks >0.2 AU. There was no significant difference in resting haemolysis levels between cohorts A and C, confirming that haemolysis at rest is prevelant in Tb racehorses in high-intensity exercise training.

## Discussion

The stability and ease of access for collecting samples for ci-miRNA measurement make them attractive as potential diagnostic biomarkers. However, in order to investigate ci-miRNAs as diagnostic tools it is vital to identify normal patterns of expression for different scenarios including responses to exercise. Furthermore, rigourous quality control measures must be in place to ensure reliable results. One adverse situation identified to cause variation in plasma ci-miRNA measurement is RBC haemolysis resulting in leakage of miRNAs into the plasma [22, 23, 33]. This is important to consider since there appears to be distinct miRNA profiles for the various cell types in blood [22]. Keeping this in mind, the use of stringent quality controls and careful sample handling has resulted in the accurate quantification of resting and post-exercise plasma ci-miRNAs from human subjects [12].

In the present study, haemolysis above the threshold for accurate quantification of ci-miRNAs was identified in the majority of the resting plasma samples as well as in all of the T_5min_ plasma samples. Intravascular haemolysis has been identified to occur in horses following racing [26], exercise on the treadmill [34–36] and after work on the gallops [27]. Exercise-induced haemolysis in horses primarily results from increased fragility of erythrocytes [37, 38], hypothesized to be due to the frequent accumulation of RBCs in the spleen [35, 37]. Changes in pH due to anaerobic exercise has also been associated with increased erythrocyte fragility [38], which also occurs in human althletes following maximal exercise [39]. It was interesting to note that in the present study even resting plasma samples had a degree of haemolysis above the threshold for accurate quantification of ci-miRNA detection [22]. We hypothesised that since the horses were in active training for 6 days of the week, which is typical for a flat racehorse training establishment, this resulted in almost daily and consistent exercise-induced insult to the RBCs resulting in persistent levels of haemolysis. Thus, the degree of plasma haemolysis and subsequent effect on miRNA abundance is important to consider when investigating plasma ci-miRNA levels in horses that are in active exercise programmes, even when evaluating pre-exercise samples.

In the present study plasma samples were analysed spectrophotometrically to determine levels of free Hb, as this is an extremely simple and accurate method to determine the extent of plasma haemolysis [22]. For the 48/52 miRNAs detected in plasma in the current study, miRNA abundance was significantly correlated with absorbance at one or both sampling time points supporting correlation between haemolysis and plasma ci-miRNA detection. Since measurement of ≥90% of the detected plasma ci-miRNAs in the current study was correlated with haemolysis, an accurate assessment of the changes in ci-miRNA abundance in response to exercise could not be made. It is believed, however, that this problem could be overcome through the quantification of exosomal ci-miRNAs (plasma miRNAs packaged in vesicles) rather than measurement of all plasma ci-miRNAs. This technique would exclude all unpackaged miRNAs which may have been leaked into the plasma from haemolysed erythrocytes.

Our research group previously demonstrated that mRNA in equine skeletal muscle responds to a single bout of exercise as well as exercise training [31, 32]. These results underpinned our interest in evaluating the effect that exercise may have on equine skeletal muscle miRNA expression. The initial profiling for equine skeletal muscle miRNA expression in the present study was carried out on horses just entering sprint training. This was based on results from previous studies in our laboratory in which Tb skeletal muscle was found to have the greatest transcriptional response to intense exercise at this training stage [31]. The 4 h post-exercise sampling time was also chosen based on previous work performed by our research group where it was identified to be the time-point exhibiting the most transcriptional activity [31]. These observations are in agreement with several human exercise studies [40–42].

In this study we observed changes in equine skeletal muscle miRNA abundance following exercise. All five of the DE miRNAs from skeletal muscle expressed at T_4_ in the present study had been identified amongst the 48 miRNAs detected from haemolysed plasma samples from horses in cohort A. Furthermore, all five are known equine miRNAs with miR-30e-5p previously reported to be expresesed in muscle in a tissue-specific manner [20]. Of great interest is that these five DE miRNAs are amongst 167 DE miRNAs reported from pre- and 30 min post-endurance exercise whole-blood samples in a group of *n* = 14 pure-breed/half-breed Arabian horses [21].

In the present study, two members of the let-7 family of miRNAs, let-7d-3p and let-7d-5p, and miR-21-5p had increased expression following exercise. The Let-7 family of miRNAs are involved in the regulation of glucose homeostasis and insulin sensitivity [43], both key processes in energy metabolism during exercise. Mir-21 has been shown to be involved in the fibrogenic pathway and the progression of Duchenne muscular dystrophy [44], so may play a role in muscle remodelling during exercise training.

Two members of the miR-30 family, miR-30b-5p and miR-30e-5p, had decreased expression post-exercise. The miR-30 family has been shown to be down-regulated in *mdx4cv* mice (models for Duchenne muscular dystrophy), with in vitro analysis indicating that miR-30 miRNAs are decreased following injury and are increased during myoblast differentiation [45]. These findings suggest that these miRNAs may play an important role in skeletal muscle growth and repair [45]. The decreased expression in response to exercise may be related to repair of exercise-induced muscle damage. The over-representation of miRNA gene targets in exercise-related KEGG pathways certainly suggests that miRNAs play a key role in the modulation of gene expression in response to exercise.

## Conclusions

In conclusion, our results indicate even resting plasma from horses in high-intensity training may not be suitable for ci-miRNA quantification due to haemolysis, although it is possible this problem could be overcome through the quantification of exosomal ci-miRNAs. However, identification of DE miRNAs in skeletal muscle indicates that modification of miRNA expression may contribute to adaptive training responses in Tbs. It is likely that additional miRNAs are differentially regulated in response to exercise but were undetectable due to the use of a human plasma panel. Future studies using a global platform such as miRNA sequencing will be required to generate a comprehensive profile of miRNAs in equine skeletal muscle at rest and following exercise. The most powerful approach to such an investigation would be an analysis of the miRNA-Seq transcriptome.

## Abbreviations

[Hb]: Haemoglobin concentration
[LA]: Lactate concentrations
AU: Absorption units
ci-miRNAs: Circulating miRNAs
*C*_t_: Cycle threshold
DE: Differentially expressed
EDTA: Ethylenediamine tetraacetic acid
HR: Heart rate
miRNAs: MicroRNAs
mRNA: Messenger ribonucleic acid
RBC: Red blood cells
RT-qPCR: Reverse transcription quantitative real-time polymerase chain reaction
T_0_: before exercise (rest)
T_4hr_: 4 h after exercise
T_5min_: 5 mins after exercise
Tb: Thoroughbreds
WD: Work day

## Footnotes

1 Polar Equine S810i, Polar Electro Ltd., Warwick, UK

2 GPSports Systems SPI10, Canberra, Australia

3 YSI 2300 stat plus, YSI UK Ltd., Hampshire, UK

4 biofluids miRCURYTM RNA Isolation Kit, Exiqon, Denmark

5 miRCURY LNA™ Universal RT microRNA PCR, polyadenylation and cDNA synthesis kit, Exiqon, Denmark

6 NanoDrop™ Bioanalyzer ND2000, Labtech, Uckfield, UK

7 Jørgen KRUUSE A/S, Langeskov, Denmark

8 Ambion, ThermoFisher Scientific Inc., Darmstadt, Germany

9 Invitrogen, ThermoFisher Scientific Inc., Darmstadt, Germany

10 RNase-free DNase, Qiagen Ltd., Manchester, UK

11 Mini-kit, Qiagen Ltd., Manchester, UK.

12 Agilent Technologies Ireland, Ltd.

## References

[1] Bartel DP (2004). MicroRNAs: genomics, biogenesis, mechanism, and function. Cell.

[2] Cai Y, Xiaomin Y, Songnian H, Yu JA (2009). Brief review on the mechanisms of miRNA regulation. Genomics Proteomics Bioinformatics.

[3] Cortez MA, Calin GA (2009). MicroRNA identification in plasma and serum: a new tool to diagnose and monitor diseases. Expert Opin Biol Ther.

[4] Chen X, Ba Y, Ma L, Cai X, Yin Y, Wang K, Guo J, Zhang Y, Chen J, Guo X, Li Q, Li X, Wang W, Zhang Y, Wang J, Jiang X, Xiang Y, Xu C, Zheng P, Zhang J, Li R, Zhang H, Shang X, Gong T, Ning G, Wang J, Zen K, Zhang J, Zhang C-Y (2008). Characterization of microRNAs in serum: a novel class of biomarkers for diagnosis of cancer and other diseases. Cell Res.

[5] Vickers KC, Palmisano BT, Shoucri BM, Shamburek RD, Remaley AT (2011). MicroRNAs are transported in plasma and delivered to recipient cells by high-density lipoproteins. Nat Cell Biol.

[6] Turchinovich A, Weiz L, Langheinz A, Burwinkel B (2011). Characterization of extracellular circulating microRNA. Nucleic Acids Res.

[7] Mitchell PS, Parkin RK, Kroh EM, Fritz BR, Wyman SK, Pogosova-Agadjanyan EL, Peterson A, Noteboom J, O'Briant KC, Allen A, Lin DW (2008). Circulating microRNAs as stable blood-based markers for cancer detection. Proc Natl Acad Sci.

[8] Reid G, Kirschner MB, van Zandwijk N (2011). Circulating microRNAs: association with disease and potential use as biomarkers. Crit Rev Oncol Hematol.

[9] Baggish AL, Hale A, Weiner RB, Lewis GD, Systrom D, Wang F, Wang TJ, Chan SY (2011). Dynamic regulation of circulating microRNAs during acute exhaustive exercise and sustained aerobic exercise training. J Physiol.

[10] Bye A, Rosjo H, Aspenes ST, Condorelli G, Omland T, Wisloff U (2013). Circulating microRNAs and aerobic fitness–the HUNT-study. PLoS One.

[11] Russell AP, Lamon S, Boon H, Wada S, Guller I, Brown EL, Chibalin AV, Zierath JR, Snow RJ, Stepto N, Wadley GD, Akimoto T (2013). Regulation of miRNAs in human skeletal muscle following acute endurance exercise and short-term endurance training. J Physiol.

[12] Nielsen S, Akerstrom T, Rinnov A, Yfanti C, Scheele C, Pedersen BK, Laye MJ (2014). The miRNA plasma signature in response to acute aerobic exercise and endurance training. PLoS One.

[13] Davidsen PK, Gallagher IJ, Hartman JW, Tarnopolsky MA, Dela F, Helge JW, Timmons JA, Phillips SM (2011). High responders to resistance exercise training demonstrate differential regulation of skeletal muscle microRNA expression. J Appl Physiol.

[14] Keller P, Vollaard NB, Gustafsson T, Gallagher IJ, Sundberg CJ, Rankinen T, Britton SL, Bouchard C, Koch LG, Timmons JA (2011). A transcriptional map of the impact of endurance exercise training on skeletal muscle phenotype. J Appl Physiol.

[15] Guescini M, Guidolin D, Vallorani L, Casadei L, Gioacchini AM, Tibollo P, Battistelli M, Falcieri E, Battistin L, Agnati LF, Stocchi V (2010). C2C12 myoblasts release micro-vesicles containing mtDNA and proteins involved in signal transduction. Exp Cell Res.

[16] Pedersen BK, Febbraio MA (2012). Muscles, exercise and obesity: skeletal muscle as a secretory organ. Nat Rev Endocrinol.

[17] Van der Kolk JH, Pacholewska A, Gerber V (2015). The role of microRNAs in equine medicine: a review. Vet Q.

[18] Barrey E, Bonnamy B, Barrey EJ, Mata X, Chaffaux S, Guerin G (2010). Muscular microRNA expressions in healthy and myopathic horses suffering from polysaccharide storage myopathy or recurrent exertional rhabdomyolysis. Equine Vet J.

[19] Gim JA, Ayarpadikannan S, Eo J, Kwon YJ, Choi Y, Lee HK, Park KD, Yang YM, Cho BW, Kim HS (2014). Transcriptional expression changes of glucose metabolism genes after exercise in thoroughbred horses. Gene.

[20] Kim MC, Lee SW, Ryu DY, Cui FJ, Bhak J, Kim Y (2014). Identification and characterization of microRNAs in normal equine tissues by next generation sequencing. PLoS One.

[21] Mach N, Plancade S, Pacholewska A, Lecardonnel J, Riviere J, Moroldo M, Vaiman A, Morgenthaler C, Beinat M, Nevot A, Robert C, Barrey E (2016). Integrated mRNA and miRNA expression profiling in blood reveals candiate biomarkers associated with endurance exercise in the horse. Sci Rep.

[22] Kirschner MB, Kao SC, Edelman JJ, Armstrong NJ, Vallely MP, van Zandwijk N, Reid G (2011). Haemolysis during sample preparation alters microRNA content of plasma. PLoS One.

[23] McDonald JS, Milosevic D, Reddi HV, Grebe SK, Algeciras-Schimnich A (2011). Analysis of circulating microRNA: preanalytical and analytical challenges. Clin Chem.

[24] Blondal T, Jensby Nielsen S, Baker A, Andreasen D, Mouritzen P, Wrang Teilum M, Dahlsveen IK (2013). Assessing sample and miRNA profile quality in serum and plasma or other biofluids. Methods.

[25] Kirschner MB, Edelman JJ, Kao SC, Vallely MP, van Zandwijk N, Reid G (2013). The impact of hemolysis on cell-free microRNA biomarkers. Front Genet.

[26] Masini AP, Tedeschi D, Badagli P, Sighieri C, Lubas C (2003). Exercise-induced intravascular haemolysis in standardbred horses. Comp Clin Path.

[27] Cywinska A, Szarska E, Kowalska A, Ostaszewski P, Schollenberger A (2011). Gender differences in exercise--induced intravascular haemolysis during race training in thoroughbred horses. Res Vet Sci.

[28] Fonseca RG, Kenny DA, Hill EW, Katz LM (2010). The association of various speed indices to training responses in thoroughbred flat racehorses measured with a global positioning and heart rate monitoring system. Equine Vet J.

[29] Dingboom EG, Dijkstra G, Enzerink E, van Oudheusden HC, Weijs WA (1999). Postnatal muscle fibre composition of the gluteus medius muscle of Dutch Warmblood foals; maturation and the influence of exercise. Equine Vet J.

[30] Vlachos IS, Kostoulas N, Vergoulis T, Georgakilas G, Reczko M, Maragkakis M, Paraskevopoulou MD, Prionidis K, Dalamagas T, Hatzigeorgiou AGDIANA (2012). miRPath v.2.0: investigating the combinatorial effect of microRNAs in pathways. Nucleic Acids Res.

[31] McGivney BA, Eivers SS, MacHugh DE, MacLeod JN, O'Gorman GM, Park SD, Katz LM, Hill EW (2009). Transcriptional adaptations following exercise in thoroughbred horse skeletal muscle highlights molecular mechanisms that lead to muscle hypertrophy. BMC Genomics.

[32] McGivney BA, McGettigan PA, Browne JA, Evans AC, Fonseca RG, Loftus BJ, Lohan A, MacHugh DE, Murphy BA, Katz LM, Hill EW (2010). Characterization of the equine skeletal muscle transcriptome identifies novel functional responses to exercise training. BMC Genomics.

[33] Pritchard CC, Kroh E, Wood B, Arroyo JD, Dougherty KJ, Miyaji MM, Tait JF, Tewari M (2012). Blood cell origin of circulating microRNAs: a cautionary note for cancer biomarker studies. Cancer Prev Res.

[34] Schott HC, Hodgson DR, Bayly WM (1995). Haematuria, pigmenturia and proteinuria in exercising horses. Equine Vet J.

[35] Hanzawa K, Higara A, Yoshida Y, Hara H, Kai M, Kubo K, Watanabe S (2002). Effects of exercise on plasma haptoglobin composition in control and splenectomized thoroughbred horses. J Equine Sci.

[36] Inoue Y, Matsui A, Asai Y, Aoki F, Matsui T, Yano H (2005). Effect of exercise on iron metabolism in horses. Biol Trace Elem Res.

[37] Hanzawa K, Kai M, Hirag A, Watanabe S (1999). Fragility of red cells during exercise is affected by blood pH and temperature. Equine Vet J.

[38] Hanzawa K, Watanabe S (2000). Changes in osmotic fragility of erythrocytes during exercise in athletic horses. J Equine Sci..

[39] Smith JA, Telford RD, Kolbuch-Braddon M, Weidermann MJ (1995). Lactate/H+ uptake by red blood cells during exercise alters their physical properties. Eur J Appl Physiol Occup Physiol.

[40] Yang Y, Creer A, Jemiolo B, Trappe S (2005). Time course of myogenic and metabolic gene expression in response to acute exercise in human skeletal muscle. J Appl Physiol.

[41] Mahoney DJ, Safdar A, Parise G, Melov S, Fu M, MacNeil L, Kaczor J, Payne ET, Tarnopolsky MA (2008). Gene expression profiling in human skeletal muscle during recovery from eccentric exercise. Am J Physiol Regul Integr Comp Physiol.

[42] Nader GA, von Walden F, Liu C, Lindvall J, Gutmann L, Pistilli EE, Gordon PM (2014). Resistance exercise training modulates acute gene expression during human skeletal muscle hypertrophy. J Appl Physiol.

[43] Frost RJ, Olson EN (2011). Control of glucose homeostasis and insulin sensitivity by the Let-7 family of microRNAs. Proc Natl Acad Sci.

[44] Ardite E, Perdiguero E, Vidal B, Gutarra S, Serrano AL, Munoz-Canoves P (2012). PAI-1-regulated miR-21 defines a novel age-associated fibrogenic pathway in muscular dystrophy. J Cell Biol.

[45] Guess MG, Barthel KK, Harrison BC, Leinwand LA (2015). miR-30 family microRNAs regulate myogenic differentiation and provide negative feedback on the microRNA pathway. PLoS One.

